# Results of an Orthopedic Veterans Affairs Medical Center Cadaveric Simulation Lab

**DOI:** 10.7759/cureus.109193

**Published:** 2026-05-19

**Authors:** Connor Ott, Torben Urdahl, George Thomas, Anthony Bitar, Christopher Chermside-Scabbo, Ramces Francisco, Jeffrey Luna

**Affiliations:** 1 Orthopaedic Surgery, University of Minnesota School of Medicine, Minneapolis, USA; 2 Orthopaedic Surgery, University of Minnesota, Minneapolis, USA; 3 Orthopaedic Surgery, Minneapolis Veterans Affairs Health Care System, Minneapolis, USA

**Keywords:** anatomy, cadaver, education, interprofessional, simulation

## Abstract

Background

This study aimed to quantitatively evaluate the quality, validity, and effectiveness of a novel orthopedic surgery cadaveric simulation program, the first published analysis of such a training model within a government-based healthcare system.

Methodology

Survey data collected immediately before, immediately following, and three months after cadaver labs assessed participant comfort with regional anatomy and procedure skills practiced during the simulations. The study was conducted at the United States Minneapolis Veterans Affairs (VA) Medical Center, a large VA hospital and teaching site for multiple health professions trainees. A total of 32 responses (29 unique individuals) were included. In total, 23 responses came from PGY1 orthopedic surgery trainees; additional participants included medical students, occupational therapy trainees, prosthetics trainees, and upper-level orthopedic trainees, most affiliated with the University of Minnesota.

Results

A total of 30 (94%) participants completed the immediate post-lab survey, and 14 (44%) completed the three-month follow-up. Significant increases in confidence were observed across all core survey items immediately after lab participation, with the largest gains in understanding surgical procedures and anticipating instrumentation needs. Confidence in anatomy comprehension and radiographic interpretation remained significantly improved at three months, while confidence in the use of technical instruments declined, possibly due to limited post-lab opportunities for continued practice. All items assessing the perceived educational value of the labs received high ratings (mean score ≥4.0 out of 5).

Conclusions

This study demonstrates the effectiveness of an orthopedic cadaveric simulation program in improving trainee confidence in anatomy, radiograph interpretation, and surgical skills. These findings support integrating similar cadaver simulation programs at other teaching centers. More frequent simulations and additional practice with surgical instruments may further enhance their educational impact.

## Introduction

Cadaveric laboratory simulation for surgical trainees has been shown to be an effective means of surgical skill acquisition and increasing surgical confidence across multiple surgical specialties [1-6]. These simulations, i.e., structured practice of real surgical procedures where cadavers are used to teach the anatomy, surgical approach, and use of instruments, are particularly useful because they provide a way to approach anatomy as it is encountered in a real patient and allow for the handling of actual tissues [4]. In orthopedic surgery, such simulations have proven beneficial in teaching operative techniques to both trainee surgeons and medical students [7,8]. Notably, cadaveric experiences have been found to offer superior educational value compared to sawbones models, a commonly used orthopedic training tool [7]. Orthopaedic trainees have found cadaveric simulation models useful to practice surgical skills in a low stakes setting [9]. Despite the identified importance of these training simulations, there remains a paucity of literature evaluating and quantifying their efficacy.

Numerous orthopedic surgery residency programs partner with government-based teaching facilities such as Veterans Affairs (VA) medical centers to provide evidence-based care while further promoting the education of orthopedic trainees [10]. VA hospitals are federally funded medical centers that provide care to veterans of the United States armed forces [10]. They are scattered throughout the United States and have a presence in nearly every major population center [10]. The VA has a long history of being a teaching institution for medical students, residents, and other health-professions trainees [10]. Cadaveric simulations at government-based teaching facilities are of interest around the world, especially in countries such as India or Sierra Leone. where medical training is largely at government medical colleges [11-15]. To our knowledge, no previously published studies have described a cadaveric simulation program within a government-based medical center.

At the Minneapolis Veterans Affairs Medical Center (VAMC), a high-volume, tertiary-care VA medical center, a novel orthopedic cadaveric simulation program has been taking place since 2016. The program was conceived to provide VA-affiliated orthopedic surgery trainees with additional operative experiences in a controlled setting. Initially focused on total joint arthroplasty, reflecting the more than 4,000 arthroplasties performed at the Minneapolis VAMC since 2016, the program has since expanded to include topics such as forearm/hand anatomy, surgical approaches to the pelvis, and more. An additional goal of the program was to further improve collegiality between professionals at the Minneapolis VAMC. Each session invites and includes surgical technicians, radiology technologists, nurses, and medical students, fostering interprofessional collaboration. During the sessions, orthopedic surgery trainees are overseen by Minneapolis VAMC staff orthopedic surgeons who provide expert advice on all aspects of the covered cases, including positioning, approaches, surgical steps, surgical technique, implant positioning, and more. The objective of this study is to quantitatively evaluate the quality, validity, and effectiveness of this novel orthopedic surgery cadaveric simulation program, providing the first published analysis of such a training model within a government-based healthcare system. This is an exploratory study using self-reported outcomes through questionnaires and does not contain a control group.

## Materials and methods

Study design

This prospective observational study was conducted at the Minneapolis VAMC between 2023 and 2025 following institutional review board approval. Eligible participants included medical students, orthopedic surgery trainees, and other medical trainees affiliated with the University of Minnesota who attended at least one cadaveric simulation lab during the study period. Participation was voluntary, and no incentives were offered. Participant recruitment was not performed directly by participants’ supervising physicians to avoid any perceived coercion or conflict of interest. Instead, study invitations were shared by study team members unaffiliated with participants’ medical training evaluations.

All willing participants were asked to complete three electronic surveys administered through Veterans Affairs REDCap: a pre-lab survey, a post-lab survey administered immediately afterward, and a second post-lab follow-up survey administered three months after. No identifying information was collected, and responses were anonymized.

Cadaver simulation lab structure

Basic demographic information was collected on the initial pre-lab survey, including age, gender, level of training, and prior experience with cadaveric simulation at the Minneapolis VAMC. All three surveys included a core set of identical questions utilizing a five-point Likert-type scale to assess self-reported confidence in surgical anatomy, procedural knowledge, operative decision-making, and postoperative evaluation. The post-lab and three-month follow-up surveys also included additional survey items designed to assess perceived educational value and longer-term retention of surgical skills and confidence. These survey items were developed internally by the residents, medical students, and attending physicians who made up the research team. The items were designed to measure participant confidence in a wide range of domains. No items were taken from previously published studies due to the lab structure and the interdisciplinary nature of the labs, which differed from those of previously published studies.

Cadaveric simulation labs were held approximately every two months and covered a variety of orthopedic topics. Several of these labs were focused on joint arthroplasty, both upper and lower extremity. Other sessions focused more on the regional anatomy of the forearm, shoulder, hip, and knee. Each session was led by at least two Minneapolis VAMC orthopedic surgeons and incorporated interprofessional participants, including medical students, trainees, surgical technicians, and other healthcare trainees. Labs varied in length based on the number of participants and procedures practiced, but averaged around two hours each. Each lab included ample time for instructors to answer participants’ questions and allowed flexibility to tailor the instruction to the participants’ trainee level. Surgical instruments and implants used during the labs were either donated to the department or consisted of expired/slightly worn instruments not appropriate for use in live operating rooms. Cadaveric specimens were funded through departmental educational funds specifically designated for trainee training.

Statistical analysis

Survey responses were aggregated and analyzed to assess changes in self-reported confidence and learning outcomes over time. Data analysis was performed by the study team. Paired t-tests were performed using R (version 4.6.0 for Mac) for each survey item that was included in the pre-lab and post-lab survey. The same analysis was performed to compare scores from the pre-lab surveys with the three-month follow-up survey. As certain items were only included in one survey, these were simply reported as a mean score.

## Results

From 2023 to 2025, 32 participants (mean age = 29 years) attended seven different cadaver dissection labs at the Minneapolis VAMC. In total, 23 (72%) participants were PGY1 orthopedic surgery trainees. Overall, 30 (94%) participants completed the post-lab survey, and 14 (44%) completed the three-month follow-up survey (Table 1). The 32 participants represented 29 unique individuals. Four of the seven labs were explicitly focused on joint arthroplasty.

**Table 1:** Descriptive statistics of conducted labs and participants.

Total number of labs	7
Upper extremity	2
Lower extremity	4
PGY1 skills week	1
Orthopedic surgery trainees	23
PGY1	13
PGY2	0
PGY3	7
PGY4	0
PGY5	3
Medical students	5
MS3	4
MS4	1
Prosthetic trainees	3
Occupation therapy trainees	1
Total participants	32
Average participant age	28.6

**Figure 1:**
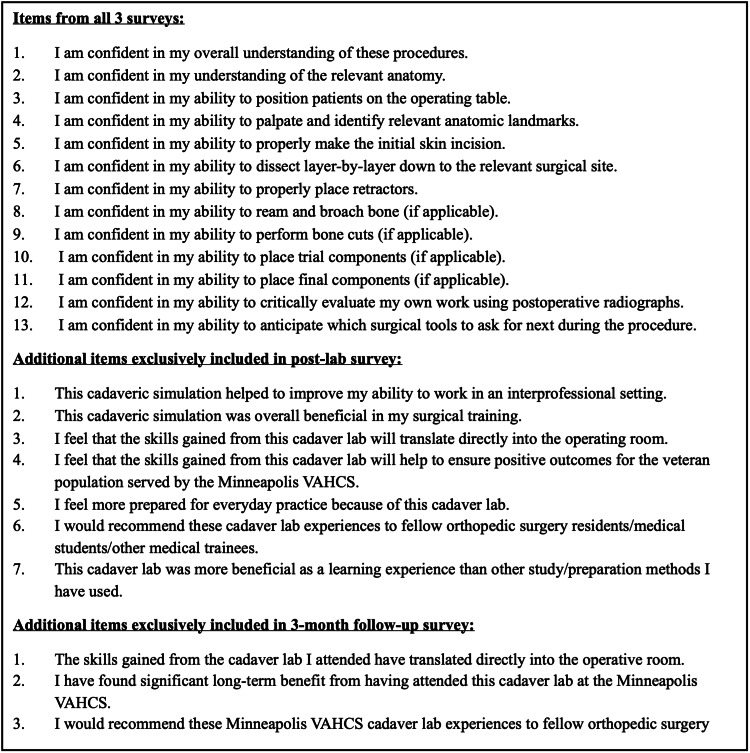
Survey items.

Average scores significantly improved for all common survey items between the pre-lab and post-lab surveys. The highest average score improvements were found in common item #1 (39% improvement in score) and item #13 (39% improvement) (Table 2). Item #1 tested confidence in overall understanding, and item #13 tested confidence in anticipation of the next surgical tool needed (Figure 1).

**Table 2:** Average respondent scores in common items of the pre-lab, post-lab, and three-month follow-up surveys. Average respondent scores (1-5 Likert scale with 1 being strongly disagree and 5 being strongly agree) for common items in post-lab surveys and three-month follow-up surveys were compared to the pre-lab survey. Paired t-tests were performed to compare pre- and post-lab scores. Bolded values indicate statistically significant differences. P-values are displayed in parentheses following the average score for each item.

Question	Average score in pre-lab survey (N = 32)	Average score in post-lab survey (N = 30)	Average score in 3-month follow-up survey (N = 14)
I am confident in my overall understanding of these procedures	3.00	4.17 (.01)	3.71 (.01)
I am confident in my understanding of the relevant anatomy	3.55	4.33 (.01)	4.21 (.01)
I am confident in my ability to position patients on the operating table	3.40	4.14 (.01)	3.77 (.02)
I am confident in my ability to palpate and identify relevant anatomic landmarks	3.71	4.5 (.01)	4.21 (.01)
I am confident in my ability to properly make the initial skin incision	3.21	4.22 (.01)	3.85 (.01)
I am confident in my ability to dissect layer-by-layer down to the relevant surgical site	2.97	3.89 (.01)	3.23 (.02)
I am confident in my ability to properly place retractors	2.79	3.79 (.01)	3 (.24)
I am confident in my ability to ream and broach bone (if applicable)	2.83	3.82 (.01)	3.1 (.71)
I am confident in my ability to perform bone cuts (if applicable)	2.88	3.78 (.01)	3.2 (.24)
I am confident in my ability to place trial components (if applicable)	3.00	3.81 (.01)	2.9 (.40)
I am confident in my ability to place final components (if applicable)	2.96	3.65 (.01)	3 (.33)
I am confident in my ability to critically evaluate my own work using postoperative radiographs	2.63	3.41 (.01)	3.2 (.02)
I am confident in my ability to anticipate which surgical tools to ask for next during the procedure	2.83	3.93 (.01)	3.08 (.14)

Average scores improved for all common survey items between the pre-lab and three-month follow-up surveys, except for item #10, which had a 0.1 decrease. Average scores significantly improved for common items #1-6 and item #12. The highest average score improvements were found in common item #1 (24%) and item #12 (22%). Item #12 tested confidence in the ability to critically evaluate postoperative radiographs.

Average scores for all common survey items decreased between the post-lab and three-month follow-up surveys. The largest average score decreases were found in item #10 (24%) and item #13 (22%). Item #10 tested confidence in the ability to place trial components. All items exclusively in the post-lab or three-month follow-up surveys had average scores greater than 4 (Table 3).

**Table 3:** Average respondent scores in items exclusive to the post-lab survey or the three-month follow-up survey.

Question	Average score
Items exclusively included in post-lab survey (N = 30)	
This cadaveric simulation helped to improve my ability to work in an interprofessional setting	4.73
This cadaveric simulation was overall beneficial in my surgical training	4.82
I feel that the skills gained from this cadaver lab will translate directly into the operating room	4.81
I feel that the skills gained from this cadaver lab will help to ensure positive outcomes for the veteran population served by the Minneapolis VAHCS	4.93
I feel more prepared for everyday practice because of this cadaver lab	4.77
I would recommend these cadaver lab experiences to fellow orthopedic surgery residents/medical students/other medical trainees	4.97
This cadaver lab was more beneficial as a learning experience than other study/preparation methods I have used	4.87
Items exclusively included in the 3-month follow-up survey (N = 14)	
The skills gained from the cadaver lab I attended have translated directly into the operating room	4.62
I have found significant long-term benefit from having attended this cadaver lab at the Minneapolis VAHCS	4.71
I would recommend these Minneapolis VAHCS cadaver lab experiences to fellow orthopedic surgery residents/medical students/other medical trainees	4.86

## Discussion

Cadaveric simulations have been shown in the literature to be an effective means of acquiring surgical skills and increasing confidence across multiple surgical specialties [1-6]. However, to our knowledge, no previous studies have described a cadaveric simulation program in a government-based healthcare system, despite training at institutions such as the VA system being a critical part of surgical education [10]. The objective of this study was to describe and evaluate the effectiveness of a novel orthopedic surgery cadaveric simulation program at the Minneapolis VAMC, the first published analysis of such a training model within the system, and one that readers interested in surgical education with access to government medical facilities could implement. The setup for one of these cadaveric simulation labs is shown in the Appendices.

Following participation in the labs, participant confidence statistically significantly increased across all assessed domains. These results indicate that the format of these labs is conducive to learning and gaining confidence for participants at various stages of training. Different aspects of the labs were more relevant for different trainees, with the anatomy review likely being more applicable for students and the technical skills practice being of more benefit to trainees. The labs also served as an opportunity to practice interprofessional communication skills. Lab participants reported that they found the labs beneficial overall and would recommend them to other trainees, further supporting the idea that these labs are a useful teaching tool. As VA medical centers are usually teaching facilities for trainees of many different levels [10], these results imply that more multidisciplinary government-based medical colleges should consider incorporating this type of lab as a teaching tool for their students and trainees.

The results from the three-month follow-up survey show that understanding of anatomy and surgical approach was still significantly better than before the labs, indicating that the format of the labs was conducive to reinforcing these skills in the longer term. Similarly, confidence in interpreting postoperative radiographs remained significantly improved three months later. This may be because knowledge of anatomy, surgical approach, and interpreting radiographs is more visually oriented and already a part of the orthopedic trainees’ day-to-day routine, giving the trainees more opportunities to practice and reinforce these skills.

In contrast to the surgical approach and interpretation of radiographs, which remained significantly improved three months later, the skills of being able to use specific instruments during the surgery, such as retractors, broaches, and trial components, did not change significantly between the pre-lab survey and the three-month follow-up survey, despite improving in the immediate post-lab survey. This is likely because these skills require more than one session to gain proficiency with, and more junior trainees do not get the opportunity to practice these skills nearly as frequently as they do interpreting radiographs. These findings mirror what a previous study using cadaver labs for teaching orthopaedic trainees found, where knowledge of surgical anatomy and confidence in the operating room were improved more than understanding of specific techniques or use of particular instruments [16]. A study of general surgery trainees and medical students similarly found that an improved knowledge of anatomy was perceived to be the biggest benefit of cadaver labs [5]. This is in contrast, however, to a similar study of podiatry trainees who found cadaver labs to be equally or more helpful for gaining confidence with instrumentation and technique as they were for familiarization with anatomy [2]. The inclusion of medical students and trainees from other healthcare professions in our study alongside orthopedic surgery trainees may partially explain this discrepancy, as the fine details of surgical technique and instrumentation were above the training level or outside the scope of practice for some participants.

An additional plausible reason why the skills of using specific instruments were less improved is that there are a vast number of different implants and manufacturers. Without a good, organized resource for referencing these instruments, it requires the trainee to have more operating experience or someone in the room with instrument-specific knowledge to feel comfortable using all the equipment. Future labs could be adjusted to allow further time to learn about and gain confidence with instrumentation, given these results. More frequent labs may have helped to improve knowledge retention across domains from the post-lab survey to the three-month follow-up, but this would come with increased costs. Future studies investigating retention rates with shorter intervals between labs may be warranted.

Our study has many limitations that limit the generalizability of our findings. Most survey participants were orthopedic interns, and future studies should investigate the utility of a cadaveric curriculum for senior trainees. Our results are vulnerable to self-selection bias as trainees were not required to participate in the cadaveric curriculum. However, the results indicate that participants do derive a benefit from the curriculum, which may inspire pilot programs that require mandatory participation. Another limitation is that most of our cadaveric sessions were focused on the lower extremity, although surgical skills from lower extremity procedures are relevant to upper extremity procedures. Finally, only 14 (44%) participants completed the three-month post-lab survey, and as with any survey study, our results are vulnerable to courtesy bias. However, the positive results of the study are reassuring that a cadaveric curriculum may be of benefit to an orthopedic trainee curriculum.

A final limitation of the study, as designed, is that it lacked a control group that did not participate in the cadaver labs. By not having a control group, it is impossible to draw firm conclusions about whether increased knowledge and comfort with procedures came from participation in the labs themselves or from additional training and experiences outside of the labs being studied. The surveys, as designed in this study, contained many items specific to individuals who participated in the labs. Future studies could be designed in such a way to more directly compare the knowledge of individuals who participated in cadaver labs to those who did not by writing survey items that were more applicable to a control group.

The costs of the acquisition of cadavers were the only costs associated with the implementation of our curriculum. Full-body cadavers at our institution cost approximately $2,800 USD per cadaver. Bilateral upper or lower extremities can cost up to $1,800 USD. For our study, all implants and instruments were donated by various implant companies. With appropriate partnership with medical device companies, the cadaveric curriculum outlined in this study is likely feasible at most institutions. Internationally, medical training is largely based at government medical colleges, like the Minneapolis VA hospital, but serving a larger population. The study shows that these institutions can similarly implement a cadaveric simulation curriculum that is of benefit to surgical trainees. Interest has grown in recent years for the use of virtual reality in surgical training, which may cost over $30,000 USD [17]. While access models to cadavers may vary by region and country, we believe a cadaveric curriculum to be more cost-effective and accessible to trainees.

## Conclusions

Our study is the first to evaluate a unique orthopedic cadaveric curriculum at a government-based teaching hospital. We found participants to have sustained benefit in their understanding of anatomy and surgical approach. Our methods serve as a model for orthopedic education programs around the world to augment their curricula. Future studies should investigate long-term results of cadaveric simulations and their impact on observed trainee surgical skills.

## Figures and Tables

**Figure 2:**
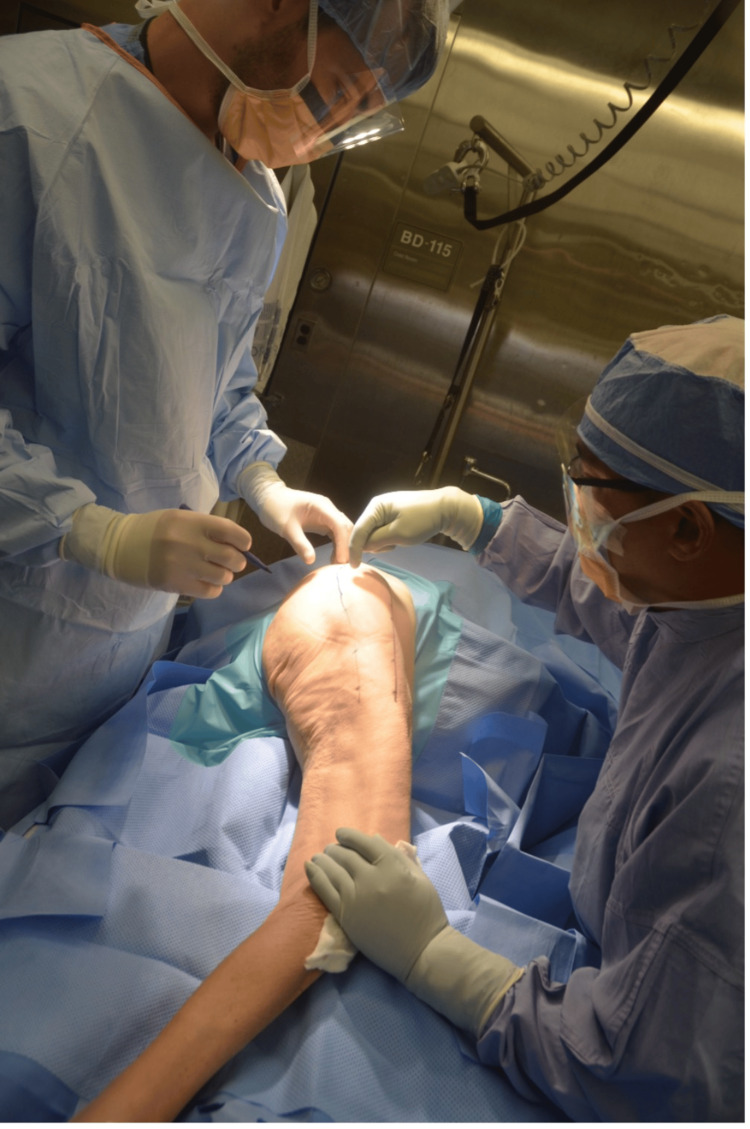
Cadaver lab dissection photo 1.

**Figure 3:**
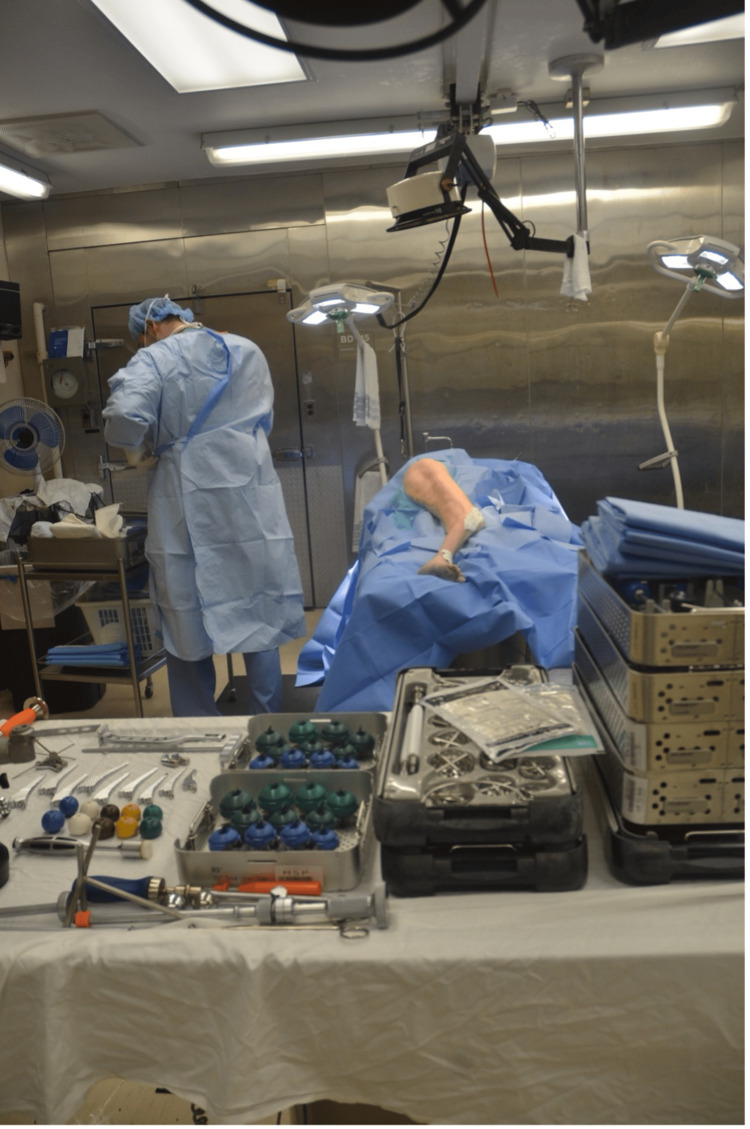
Cadaver lab photo 2.
