# Correction: Results of an Orthopedic Veterans Affairs Medical Center Cadaveric Simulation Lab

**DOI:** 10.7759/cureus.c449

**Published:** 2026-06-10

**Authors:** Connor Ott, Torben Urdahl, George Thomas, Anthony Bitar, Christopher Chermside-Scabbo, Ramces Francisco, Jeffrey Luna

**Affiliations:** 1 Orthopaedic Surgery, University of Minnesota School of Medicine, Minneapolis, USA; 2 Orthopaedic Surgery, University of Minnesota, Minneapolis, USA; 3 Orthopaedic Surgery, Minneapolis Veterans Affairs Health Care System, Minneapolis, USA

A sentence in the Introduction section has been corrected to: “Orthopaedic trainees have found cadaveric simulation models useful to practice surgical skills in a low stakes setting.” The earlier version of this article misstated a training requirement for orthopedic trainees according to ABOS guidelines.

